# Risk Factors for Hepatitis C Virus Transmission Obscure in Nigerian Patients

**DOI:** 10.1155/2011/939673

**Published:** 2011-07-13

**Authors:** Olive Obienu, Sylvester Nwokediuko, Abraham Malu, Olufunmilayo A. Lesi

**Affiliations:** ^1^Gastroenterology Unit, Department of Medicine, University of Nigeria Teaching Hospital, Ituku/Ozalla, PMB 01129 Enugu, Nigeria; ^2^Department of Medicine, Jos University Teaching Hospital, Jos, Nigeria; ^3^Department of Medicine, Lagos University Teaching Hospital, Lagos, Nigeria

## Abstract

*Aim*. To determine the prevalence of anti-HCV and risk factors associated with HCV infection in Nigerians. *Materials and Method*. Patients attending a general outpatient clinic were administered a structured questionnaire on the risk factors for HCV infection. They were also tested for anti-HCV using a third generation enzyme-linked immunosorbent assay. *Result*. The seroprevalence of anti-HCV was 4.7%. Among the risk factors evaluated, none was found to be significantly associated with anti-HCV seropositivity. *Conclusion*. The risk factors associated with HCV infection in Nigerian patients are obscure. This warrants further studies on the epidemiology of this important cause of liver disease.

## 1. Introduction

Hepatitis C virus (HCV) infection continues to be a major disease burden on the world. In 1999, the World Health Organisation (WHO) estimated a worldwide prevalence of about 3% with the virus affecting 170 million people worldwide [1]. A more recent estimate puts the prevalence at 2.2% corresponding to about 130 million HCV-positive persons worldwide [2]. Because many countries lack data, this estimate is based on weighted averages for regions rather than individual countries.

In the industrialized countries, HCV accounts for 20% of cases of acute hepatitis, 70% of cases of chronic hepatitis, 40% of cases of end stage cirrhosis, 60% of cases of hepatocellular carcinoma (HCC), and 30% of liver transplants [3, 4]. An estimated 27% of cirrhosis and 25% of hepatocellular carcinoma worldwide occur in HCV-infected people [5].

The most efficient transmission of HCV is through large or repeated direct percutaneous exposure to blood (of transfusion or transplantation from infectious donors and injecting drug use) [6]. HCV is less efficiently transmitted by single small-dose percutaneous exposure (e.g., accidental needle sticks) [6, 7] or by mucosal exposures to blood or serum-derived fluids (e.g., birth to an infected mother and sexual intercourse with an infected partner) [6, 8, 9]. The high transmission rate through illicit intravenous drug use (IVDU) explains why the prevalence of HCV among people who acquired human immunodeficiency virus (HIV) through IVDU reaches 90% [10].

The risk factors for HCV transmission in Nigeria have not been properly characterized. Even in the western world where some risk factors have been identified, recent studies indicate that the global epidemiology of HCV has been changing [11, 12]. This scenario has compounded efforts at developing strategies for HCV screening. This study was designed to determine the prevalence of HCV infection in Nigerian patients and to determine the risk factors associated with it.

## 2. Materials and Methods

This study was a cross-sectional seroprevalence study involving adult patients attending the General out-patient clinic of the University of Nigeria Teaching Hospital, Ituku-Ozalla. The study was approved by the Hospital Research Ethics Committee, and the participants gave informed consent.

Patients who had symptoms and signs suggestive of liver disease were excluded. Each participant was administered a structured questionnaire containing the putative risk factors for HCV transmission.

Venous blood (5 mLs) was obtained from the participants and tested for antibody to HCV (anti-HCV) using a third generation enzyme-linked immunosorbent assay (ELISA) manufactured by DRG International Inc., USA. This test kit has a sensitivity of 95% and specificity of 97.5%.

The data was analyzed and the results expressed as means and proportions. Differences between means and proportions were determined using student's *t*-test and chi-squared test, respectively. A *P* value of ≤0.05 was considered statistically significant.

## 3. Results

Three hundred and sixty (360) patients participated in the study. They consisted of 183 males (50.8%) and 177 females (49.2%). Their ages ranged between 19 years and 75 years (mean = 36.4 ± 9.4 years). Seventeen patients tested positive for anti-HCV (4.7%). The mean age of the anti-HCV-positive patients was 36.0 ± 8.1 years while the mean age of the anti-HCV-negative patients was 36.8 ± 9.9 years. The difference between the means was not statistically significant (*P* = 0.9526).  Table 1 illustrates the anti-HCV serostatus and gender distribution of the patients. The putative risk factors were marginally more frequent in the anti-HCV seropositive patients compared to the anti-HCV-negative group, but none reached statistical significance (Table 2).

## 4. Discussion

The seroprevalence of anti-HCV in this study was 4.7%. This finding is comparable to the results of similar studies across the continent [13–21]. However, none of the putative risk factors evaluated in this study showed any significant association with anti-HCV seropositivity.

Transfusion-associated HCV infection was a predominant worldwide risk before HCV testing became available. It has been virtually eliminated in those countries that implemented routine HCV testing of donors [22], but in others, receipt of blood transfusion remains an important source of infection. Some countries continue to use commercial donors to supplement their blood supplies [23]. In Nigeria, the low risk of transmission through blood transfusion may be related to the low prevalence of HCV in the general populace, coupled with the fact that Nigeria is successfully implementing a national blood safety programme led by Safe Blood for Africa Foundation (SBFA) to fight the spread of HIV/AIDS [24]. An estimated 3.6% of Nigerians are living with HIV and AIDS [25], which translates into about 3.3 million people. HCV coinfection with hepatitis B virus (HBV) and/or HIV has been described in some studies in Nigeria [26, 27].

Certain high-risk behaviours and practices which are prevalent in African societies have been shown to play some role in Hepatitis B virus (HBV) transmission. These include scarification marks, sharing of sharp body-piercing instruments like razor blades, sharing of toothbrushes, and instruments for native uvulectomies [28]. Uvulectomy is practiced in Nigerian communities as treatment for sore throat. It is usually carried out by local healers in very unhygienic environments without any consideration for infection control. The instruments used are not usually sterilized. The procedure carries other risks like bleeding, anemia, sepsis, and transmission of other pathogens including HIV and HBV. Deaths have resulted from such procedures [29]. 

These high-risk behaviours and practices do not seem to contribute significantly to the spread of HCV. The reason for this may also be related to the low prevalence of HCV in the populations involved. Another reason is the fact that HBV is approximately 10 times more infectious than HCV [30, 31].

The risk factors for HCV infection may be unidentified in a significant proportion of patients in many parts of the world. In a study in Iran, the risk factors for infection could not be identified in 20% of cases [32]. In other studies, risk factors accounting for infection remain unknown in 10–40% of patients with acute or chronic Hepatitis C [33, 34]. A study conducted recently at a Northern California Liver centre between 2001 and 2008 showed that Asian-Americans are more likely to present with unidentifiable risk exposure [35]. The researchers concluded that the commonly known risk factors for HCV may be more appropriate for risk assessment for Caucasians and Hispanics but not for Asian-Americans. These findings pose major implications for developing strategies for HCV screening in our increasingly culturally diverse population.

In conclusion, the seroprevalence of anti-HCV in a population of Nigerians attending a general out-patient clinic is 4.7%. The risk factors associated with anti-HCV seropositivity are largely obscure, and this calls for more studies into the epidemiology of HCV infection in order to develop effective strategies for screening.

## Figures and Tables

**Table 1:** Anti-HCV serostatus of patients.

Group	Anti-HCV positive (%)	Anti-HCV negative (%)	Total
Males	6 (3.3)	177 (96.7)	183
Females	11 (6.2)	166 (93.8)	177
Total	17 (4.7)	343 (95.3)	360

**Table 2:** Risk factors for HCV infection.

Risk factor	Anti-HCV positive (*n* = 17)	Anti-HCV negative (*n* = 343)	Relative risk	Odds ratio	*χ* ^2^	*P* value
Blood transfusion	2	36	1.115	1.121	0.022	0.882
Uvulectomy	2	37	1.46	1.495	0.013	0.91
Tattooing	0	2	0.000	3.93	0.99	0.75
Multiple sexual partners	3	59	1.025	1.026	0.002	0.97
Sharing of toothbrush	4	78	1.033	1.035	0.004	0.95
Sharing of razor blades	5	101	1.000	1.000	0.000005	1.00
Sharing of shaving sticks	1	19	1.059	1.062	0.003	0.95
Injections from quacks	2	40	1.008	1.009	0.00013	0.99
Scarification markings	1	17	1.176	1.187	0.026	0.87
Occupational exposure	0	5	0.000	1.784	0.248	0.62
Intravenous drug use	0	0	—	—	—	—
